# Willing but Unable: Moral Distress and Burnout in Italian Veterinarians Working with Companion and Farm Animals

**DOI:** 10.3390/ani14243691

**Published:** 2024-12-20

**Authors:** Emanuela Prato-Previde, Barbara De Mori, Nicoletta Colombo, Annalisa Pelosi

**Affiliations:** 1 Department of Pathophysiology and Transplantation, University of Milan, 20133 Milan, Italy; 2 Department of Comparative Biomedicine and Food Science, University of Padova, 35020 Padova, Italy; barbara.demori@unipd.it; 3 Independent Researcher, Via Po 2, 26028 Cremona, Italy; nicoletta.colombo.vet@gmail.com; 4 Department of Medicine and Surgery Neuroscience Unity, University of Parma, 41122 Parma, Italy; annalisa.pelosi@unipr.it

**Keywords:** veterinary practice, moral distress, burnout, empathy, companion animals, farm animals, animal caring

## Abstract

Veterinarians face ethical challenges during their careers, but despite evidence of work-related stress and burnout in veterinarians, moral distress has been poorly investigated. Using an online survey, professional experience, moral distress, and burnout were investigated in 704 Italian veterinarians caring for companion animals, farm animals, or both. The reliability of the moral distress questionnaire was optimal, and Factor analysis identified four factors for moral distress: I. Conflict with owner/farmer; II. Relationships with colleagues/superiors; III. Legal context/unsupportive Professional Institutions; IV. Unshared requests for euthanasia. Veterinarians reported rather low levels of moral distress but high levels of work-related stress/anxiety and a decline in empathy over time, mainly toward owners/farmers. More than 50% of veterinarians reported medium/high levels of burnout, and client-related burnout was significantly higher than animal-related burnout. Levels of work-related stress, moral distress, and burnout were higher in women, and experience exerted a protective effect. Moral distress was positively correlated with emotional exhaustion and depersonalization and, to a lesser extent, lower professional fulfillment. The relatively low level of moral distress that emerged in the study needs further investigation, especially considering the high levels of work-related stress and anxiety reported by veterinarians. Women’s greater sensitivity to moral distress and burnout should not be overlooked given the progressive feminization of the veterinary profession worldwide.

## Simple Summary

Veterinary practice is characterized by ethical conflicts between protecting animals’ health and welfare and fulfilling the requirements and interests of other people (owners, breeders, farmers, and other colleagues), without violating their own professional obligations and moral integrity. Ethical conflicts can lead veterinarians to a form of distress called “moral distress” and may favor burnout. We investigated moral distress and burnout in 704 Italian veterinarians caring for companion animals, farm animals, or both. Veterinarians reported rather low levels of moral distress, high levels of work-related stress/anxiety, and a decrease in empathy over time, mainly toward clients (owners, farmers). More than 50% of veterinarians reported medium/high levels of burnout, and client-related burnout exceeded animal-related burnout. Work-related stress, moral distress, and burnout were greater in women, and experience had a protective effect. Moral distress was positively correlated with emotional exhaustion and depersonalization and with lower professional fulfillment. The relatively low level of moral distress that emerged in this study needs further investigation, especially considering the high levels of work-related stress and anxiety reported by veterinarians. Women’s sensitivity to moral distress and burnout should not be overlooked given the progressive feminization of the veterinary profession worldwide.

## 1. Introduction

Veterinarians’ work is complex and demanding and characterized by at least four core aspects: the animals’ needs, people’s requests/interests, the profession’s obligations, and the expectations of society [1,2,3,4]. The scenario in which veterinarians practice today is characterized by conflicts of interest between protecting the health and welfare of animals and the need to fulfill the requirements and interests of other people (owners, breeders, farmers, and other colleagues), without violating their own professional obligations and moral integrity [2,5]. Conflictual situations could be considered an unavoidable part of veterinary practice but may represent a significant source of distress that can severely affect personal and professional well-being, being an additional source of stress [5,6,7,8,9].

As in human healthcare professionals [10,11], animal care professionals face both situations in which they are unsure about the best course of action, with no obvious way to prioritize one action over another, and situations in which they know what would be the “right thing to do” but are unable to implement it adequately due to internal or external constraints [7,12,13].

The latter situations are specifically characterized as moral conflicts where value-related demands are not respected. Moral conflicts are attributable to limited control or autonomy over decision-making, have been defined by vets by the phrase “I would like to, but I can’t” [13], and can be a source of distress for veterinarians, leading to a form of distress called “moral distress”.

The term moral distress was initially coined by Jameton [14], to define the negative feelings experienced by nurses “when one knows the right thing to do, but institutional constraints make it nearly impossible to pursue the right course of action” (p. 6). Moral distress is rooted in the individual’s inability to act according to his/her values and perceived obligations due to internal and external constraints [11,14,15]; it covers a variety of situations in which morally appropriate courses of action are not taken due to limited control or autonomy over decision-making (e.g., lack of time, reticence from colleagues, superiors and supervisors, power structures, legal issues, and so on).

In human healthcare professions, moral distress can lead to the development of burnout [11,16,17]. Burnout is defined as a psychological syndrome arising in response to chronic emotional and interpersonal stressors on the job and has been described as a combination of emotional exhaustion, depersonalization, and low personal accomplishment [18,19,20].

Burnout has been related to various factors, including impotence related to hierarchical power structures, ineffective or obstructive policies, dysfunctional communication patterns, lack of resources, and other issues beyond the individual’s control [15].

Studies on burnout in veterinary practice highlight its negative effects on veterinarians who may show a high level of job stress and compromised psychological well-being [21,22], leading to substance use [23], depression, and suicidal ideation [24,25].

Moral distress and its causes and consequences have received attention in human healthcare professions [11,26], and persistent moral distress is considered a major cause of depression and burnout and seems to lead to indifference in morally challenging situations [16,17,18,19,20,21,22,23,24,25,26,27]. However, despite the growing interest in animal healthcare professionals’ well-being and mental health and the evidence of occupational stress and burnout in veterinary practice [28,29,30,31], moral distress related to ethical conflicts has been scarcely investigated [4,6,7,9,13].

Differently from human healthcare professionals, veterinarians are confronted with a unique source of conflicts as they care for different species in a variety of different contexts of the human–animal relationship, facing highly different expectations from their clients regarding the treatment of their patients (i.e., pet owners, lab scientists, breeders, farmers); thus, their job is special and morally challenging, and moral distress could even be increased if they are not aware of the social conditionality of their responsibility [32,33].

Professional activities of veterinarians working with companion animals and farm animals are mainly described as a triangular affair and are morally challenging, requiring them to make difficult decisions and weigh the interests of the animals and the needs and requests of the clients, colleagues, and finally, other possible parties involved [1,34].

A widespread source of moral stress for companion animal veterinarians is the request for euthanasia for futile reasons or economic motives [6,7,35,36,37]; other sources are euthanasia of sick animals, animal abuse, being pressed to continue treatments despite really compromised animal welfare, dealing with colleague incompetence, conflict of opinions between colleagues, providing unnecessary and futile treatments, and inappropriate provision of medications and certificates [38,39,40]. Furthermore, the client’s economic constraints that interfere with the appropriate care for animals, and situations in which best patient care comes into conflict with what is best for the organization, the patient’s owners, or other patients are typical among vets working with companion animals [6,12,40].

For veterinarians working in the field of livestock farming, particularly in intensive animal production, conflicts between the interests of the animal patient and those of the clients may be even more remarkable as, at least in today’s Western societies, attitudes toward current animal husbandry and the level of animal welfare tend to be rather ambivalent [41], oscillating between an increasing concern for animal welfare and criticism about modern animal farming [42,43,44] and economic interests and consumers’ demands for cheap food prices regardless of animal health conditions [45]. Morally challenging situations are reported to be mainly related to poor husbandry conditions, conflicts between medical and economic needs, the necessity to weight up the farmers’ business considerations against animal welfare issues, and the overall context in which livestock farming takes place (e.g., legal requirements/regulations, other veterinarians and employees in various roles, and an uninformed and over-critical society [13]).

The first aim of this study was to investigate moral distress in Italian companion and farm animal veterinarians to evaluate similarities and differences in its frequency, intensity, and nature. Previous studies have considered either companion animal practitioners [6,46] or farm animal veterinarians [4,13] using different methods and surveys.

The second aim was to evaluate the relationship between moral distress and burnout in the three dimensions of the Maslach Burnout Inventory [47]. Moral distress and burnout appear to be related phenomena in human care providers and could have similar determinants [16,48,49], with burnout being one of the most damaging consequences of moral distress [15,49].

Since veterinarians are confronted with animal patients and human clients, we developed a questionnaire for assessing their professional experiences and moral distress and a modified version of the Italian MBI scale [50] to evaluate burnout levels and the role of the human and animal components on the three different burnout dimensions.

Finally, the possible relationship between moral distress, empathy erosion, and indifference in morally challenging situations was considered.

## 2. Materials and Methods

### 2.1. Participants

A total of 781 Italian veterinarians responded to an online survey made available for compilation on Forms (Office 365) from January 2022 to April 2022. The administration procedure ensured complete anonymity, and data were collected from volunteer veterinarians only after they signed an informed consent. The study was approved by the Ethical Committee of the University of Milan (n° 112/21). Individuals eligible for inclusion in the survey were veterinarians working with companion animals or farm animals or with both types of animals and with at least one year of practice. Participants were recruited with the collaboration and sponsorship of FNOVI (Federazione Nazionale Ordini Veterinari Italiani), who e-mailed the announcement and the link to the survey to all its associates, inviting them to participate in the study. Participants were also recruited by sending the link to the survey via e-mail to all personal contacts and diffusing it on social media. The use of an online survey with the collaboration and sponsorship of FNOVI allowed potential participants living all over Italy to take part in the study.

### 2.2. Questionnaire

The questionnaire was in Italian and was implemented based on the existing literature on moral distress in veterinary and human care professions [6,7,51,52]. It comprised four sections (see Supplementary Materials). In the first section, socio-demographic data (age, gender, family composition, past/present domestic animals) were collected. In the second section, participants were asked to provide some work-related information: years in veterinary practice, specialty within veterinary medicine, working context and region, collaborators at the workplace, and type of animals cared for. In the third section, participants were asked to respond to 32 items on their professional experience; most items regarded morally distressing situations during professional practice and focused on aspects considered to be central to the phenomenon [51]. Most of the items evaluating moral distress were the same for all veterinarians, and only a few were directed to vets caring just for companion or farm animals. Participants were asked to rate how often each situation (item) occurred in their practice on a Likert scale, with 0 = never, 4 = very often, and then they were asked how distressing it was when it occurred, with 0 = none, 4 = extreme distress. For each item, the frequency score was multiplied by the distress score to obtain a composite score, ranging from 0 to 16, indicating the level of moral distress [51,52]. Composite item scores were summed to create an overall score, and higher scores indicated higher levels of moral distress.

Two items evaluated participants’ feelings of anxiety/distress for their work in general (i.e., feeling stressed and anxious because of work) on a scale ranging from 0 (not at all) to 4 (extremely), and three items assessed vets’ perceived changes in compassion/empathy toward clients/colleagues and animal patients on a scale ranging from 0 (not at all) to 4 (extremely).

In the last section, participants were asked to fill up the Italian version [50] of the Maslach Burnout Inventory (MBI [47]), a 22-item self-report questionnaire that evaluates three domains of burnout: emotional exhaustion (EE—9 items), depersonalization (DP—5 items), and personal accomplishment (PA—8 items). The emotional exhaustion subscale assesses a person’s feelings of being emotionally drained and exhausted by work; the depersonalization subscale measures one’s insensitive and impersonal responses toward the recipients of his/her service, care, or treatment; finally, the personal achievement subscale assesses a person’s feelings of competence and success in working with people. Subjects respond to each item on a 7-point scale (0–6) reporting the frequency with which each statement has been recently experienced (0 = never, 6 = every day). As veterinarians interact with both animals and people, in the study, we administered an ad hoc modified version of the MBI scale (MBI-VET), in which all the items referring to relationships were split in two forms, one relative to the vet-animal interaction (MBI-VAI) and the other relative to the vet-person (owner/farmer) interaction (MBI-VPI). For example, item 6 of the MBI scale states that “Working with people the whole day is stressful for me” and was also worded as “Working with animals the whole day is stressful for me” (MBI-VAI item 6.1). This aimed to investigate the relative impact of interacting with owners/farmers versus animals, which is an essential aspect of the veterinary profession.

### 2.3. Statistical Analysis

All data were automatically uploaded from the Forms platform into a computer database (Microsoft Excel for Microsoft Office 11) for analyses. Descriptive analysis was carried out using frequencies and percentages for categorical variables and mean and standard deviation (SD) and ranges for quantitative variables. The association between categorical variables was estimated with the chi-square test. Linear models (ANOVA and linear regressions) were performed to evaluate the significance of categorical and continuous independent variables’ effect on the measure of interest. Specification tests were performed on the models’ residuals, and the appropriate corrections were adopted in case of violation (for example, Greenhouse–Geisser correction for sphericity in repeated measures ANOVAs or Welch correction for heteroscedasticity). Analysis of covariance (ANCOVA) allowed us to control the effect of disturbance variables. Zero-order and first-order correlations (Pearson’s r coefficient) were conducted to test the covariation relationships between continuous variables and to exclude spurious relationships. The false rate discovery error was checked by applying the Holm correction to pairwise comparisons between means (post hoc analysis) and to the correlation matrix. To estimate the latent factors underlying the correlation matrix of moral distress items, an exploratory factor analysis (EFA) was conducted (principal axis factorization, Maximum Likelihood method, Promax oblique rotation), choosing the best factorial solution based on the relative (Tucker Lewis Index—TLI) and absolute (Root Mean Square Residuals—RMSR, Root Mean Square Error of Approximation—RMSEA) indexes of fit. The significance threshold was set for all analyses at alpha = 0.05; however, since the large sample size can easily induce statistical significance associated with interpretative irrelevance, the inferential tests were also interpreted considering the adequate effect size coefficient. All statistical analyses were performed using R software (version 4.3.0).

## 3. Results

### 3.1. Demographic Information

Of the 781 veterinarians who responded to the survey, 77 were excluded as they did not respond to several items. Thus, the final sample consisted of 704 veterinarians: 493 women (70.3%), 205 men (29.12%), and 6 non-respondents. The participants’ average age was 43.5 years (±12, range 25 (N = 3)–73 (N = 1) yrs.). Women were younger than men (41.8 ± 11.1 vs. 47.6 ± 13.6; t318.5 = −5.43, *p* < 0.001; d = −0.493) and, consistently, had fewer years of practice on average (14.7 ± 10.8 versus 20.4 ± 13.4; t314.3 = −5.34, *p* < 0.001; d = −0.488). Most respondents lived in two-person (30.6%) or three-person (27.1%) households (including the respondent), followed by four-person households (20.4%); only 14.8% of participants lived alone, and 7.1% lived in 5- or 6-person households. Women and men were living in households of equal size (2.7 ± 1.2 versus 2.9 ± 1.2, d = −0.17), and the number of cohabitants was not correlated with age (r = 0.071). As regards animal ownership, 86.2% of participants grew up with animals, and 82.4% currently lived with an animal (mainly cats and dogs); the association between growing up and living with animals was significant and discrete (χ^2^_1_ = 26.8, *p* < 0.001; C = 0.273). As regards work, participants had worked on average for 16.4 (±11.9) years, ranging from a minimum of 1 year (N = 22) to a maximum of 50 yrs. (N = 1). Years of practice were almost perfectly correlated with age (r = 0.969) and thus were selected for subsequent analyses. Of the respondents, 64.2% declared having a post-graduate degree or specialty within vet medicine, with no differences between women and men (64% and 64.7%, respectively) and no difference between specialized and not specialized with respect to years of practice (16.8 ± 11.1 versus 16.1 ± 12.3). Most participants stated they worked in Northwest (65.0%) and Northeast (22.7%) Italy, with a minority practicing in Central (7.2%) and South (3.3%) Italy and the Islands (1.7%). More than half of respondents (51.2%) reported they never changed their workplace or mode of work, and 17.7% did so only once. Of the 704 respondents, 68.4% reported having collaborators, and the presence of collaborators was greater among companion animal vets (74.8%) compared to farm animal vets (53.2%) and companion/farm animal vets (48.6%). Most veterinarians (72.4%, N = 510) worked with companion animals exclusively and mostly with dogs and cats; 17.6% (N = 124) reported they worked just with farm animals (cattle, pigs, goats, sheep, and poultry), and 9.9% (N = 70) reported working with both animal types but predominantly with farm animals and only occasionally with companion ones (mainly dogs and cats). Women were significantly over-represented in the companion animal professionals (82.9%) and under-represented in the other two categories (farm animals, 9.9%; companion and farm, 7.1%); conversely, men were significantly over-represented in the farm animal category (36.6%).

### 3.2. Questionnaire: Moral Distress

**Figure 1:**
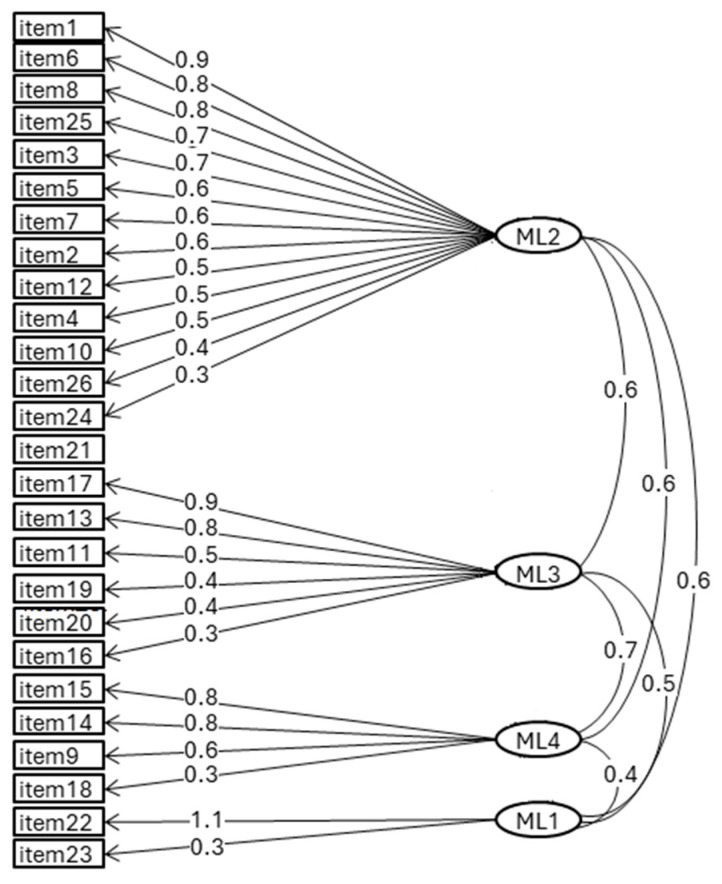
Moral distress factors: The four factors extracted through factor analysis: ML1 = Unshared euthanasia; ML2 = Conflict with owner/farmer; ML3 = Relationships with colleagues/superiors; ML4 = Legal context/unsupportive Professional Institutions.

The factorial structure and reliability of the moral distress questionnaire were verified through factor analysis, and single items were analyzed descriptively. The questionnaire reliability was optimal (Cronbach alpha = 0.92). The EFA showed that a four-factor solution had the best fit indices (RMSEA = 0.0.48, RMSR = 0.0; TLI = 0.923—should be > 0.90). The four extracted factors were labeled as follows: I. Conflict with owner/farmer (13 items); II. Relationships with colleagues/superiors (5 items); III. Legal context/unsupportive Professional Institutions (4 items); IV. Unshared requests for euthanasia (2 items). Item loadings are depicted in Figure 1. For each item, mean scores were calculated for frequency and intensity, and the level of moral distress was the composite score (“f × i”), ranging from 0 to 16 (Table 1).

**Figure 2:**
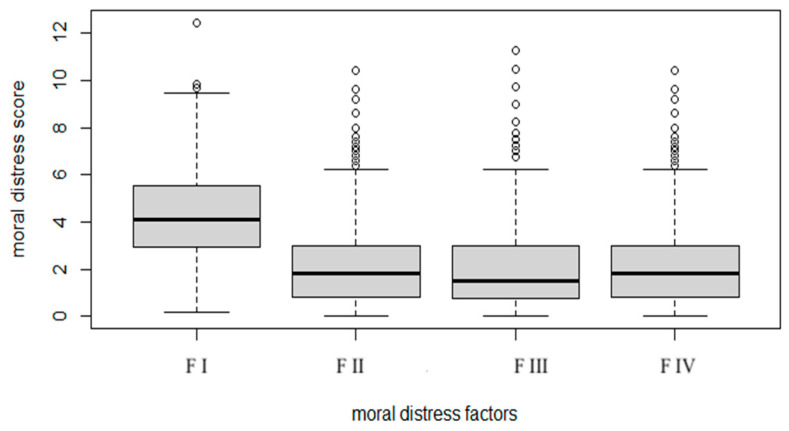
Moral distress factors and scores. FI: Conflict with owner/farmer; FII: Relationships with colleagues/superiors; FIII: Legal context/unsupportive Professional Institutions; FIV: Unshared requests for euthanasia. Dots represent outliers.

Overall, moral distress scores were quite low, with a slight prevalence of distress related to the conflictual relationship with the owners (Figure 2). The total score (the sum of the overall scores of all items included in the factor analysis) was 86.4 ± 42.1 (median = 81, min = 3, max = 282), within a theoretical range from 0 to 416; the variable was normally distributed (skewness = 0.68. kurtosis = 0.78).

The type of animal cared for had a slight but significant effect (F [2,701] = 10.1, *p* < 0.001, R2 = 0.028); the moral distress of veterinarians treating only companions (89.9 ± 41.1) and companions plus farm animals (86.7 ± 46.9) were not significantly different (*p* = 0.545, d = 0.07), whereas the moral distress of veterinaries treating only farm animals (71.3 ± 39.7) was significantly lower (companions’ vets: *p* < 0.00, d = 0.46; companions’ and farm vets: *p* = 0.027, d = 0.37). This effect was significant even if included in an analysis of covariance (ANCOVA; F [2,691] = 9.44, *p* < 0.001), partializing the years of experience effect, which was negatively and significantly correlated with moral distress (F [1,693] = 114.5, *p* < 0.001, b1 = −1.31, R2 = 0.142). However, when considering the effect of gender in the ANCOVA, its significance declined (F [2,694] = 2.83, *p* = 0.059); the effect of gender on moral distress was quite strong and significant (F [1,697] = 63.7, *p* < 0.001, d = 0.66), with women perceiving a higher level of moral distress (94.3 ± 39.5 vs. 67.5 ± 42.13) regardless of the type of animal cared for.

### 3.3. Questionnaire: Work-Related Stress and Anxiety

**Figure 3:**
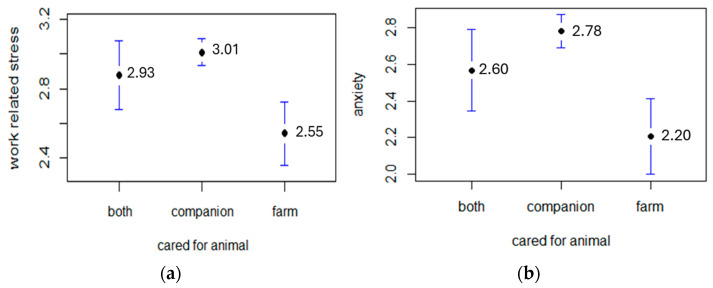
Work-related stress and anxiety. Work-related stress in relation to the type of animal cared for (**a**); work-related anxiety in relation to the type of animal cared for (**b**).

The items assessing anxiety and distress for the work in general (“Have you ever felt anxious because of your work?”; “Have you ever felt distressed because of your work?”) showed that veterinarians’ anxiety and work-related stress were relevant (2.58 ± 1.1 and 2.85 ± 0.94, respectively) and strongly correlated (r = 0.748, *p* < 0.001, 95%CI [0.713, 0.779]). A total of 26.6% of veterinarians reported being moderately job-stressed, 34.9% to be very stressed, and 32.3% stated they were highly stressed; only 5.3% said they were not or just slightly stressed. Regarding job anxiety, 27% of veterinarians reported being extremely anxious, 29.8% very anxious, and 28.1 anxious (Figure 3a,b). Professional experience had a significant protective impact on both stress (b1 = −0.02, 95%CI [−0.032, −0.021]; R2 = 0.121) and anxiety (b1 = −0.02, 95%CI [−0.031, −0.017]; R2 = 0.069).

Gender seemed to exert a weak effect on stress (F [1,692] = 25.97, *p* < 0.001; R2 = 0.036) and anxiety (F [1,696] = 38.78, *p* < 0.001; R2 = 0.053): in both cases, women scored higher than men (stress: 3.1 ± 0.88 versus 2.6 ± 1.1; anxiety: 2.8 ± 1.1 versus 2.3 ± 1.1). The gender effect, once controlled for that of professional experience (ANCOVA), was still significant, but even weaker (stress: F [1,682] = 10.78, *p* = 0.001, anxiety: F [1,685] = 22.86, *p* < 0.001).

Also, the type of animals cared for had a weak effect on stress [F (2,697] = 12.49, *p* < 0.001; R2 = 0.035) and anxiety (F [2,701] = 0.675, *p* = 0.412). Companion animals’ vets were significantly more anxious and stressed than farm vets (*p* < 0.001 for both comparisons, Figure 3a,b) and, to a lesser extent, than vets dealing with both typologies (stress: *p* = 0.013, anxiety: *p* = 0.025). No significant differences emerged between farm and companion plus farm vets (stress: *p* = 0.478, anxiety: *p* = 0.184).

### 3.4. Questionnaire: Emotional Detachment

Emotional detachment from work (How often do you feel that you are “just” doing your job?) appeared less intense than anxiety and work-related stress (1.65 ± 1.1). A total of 46.6% of respondents experienced it a little or not at all, but over 50% reported that they experienced it “quite a bit.” Only 39.1% reported little or no stress for this emotional condition. No gender differences emerged, but women were more distressed by this condition (detachment: F [2,690] = 1.25, *p* = 0.263, R2 = 0.002; stress: F [2,604] = 5.04, *p* = 0.025, R2 = 0.008). Years of professional experience exerted a significant protective impact on emotional detachment and related stress (detachment: b1 = −0.023, F [1,687] = 44.30, *p* < 0.001, R2 = 0.061; stress: b1 = −0.02, F [1,602] = 34.27, *p* < 0.001, R2 = 0.054). No significant differences in emotional detachment in relation to the type of animal cared for emerged (F [2,695] = 0.711, *p* = 0.491, R2 = 0.002).

Emotional detachment seemed associated more to a decrease in empathy toward animals, and only weakly to a decrease in empathy toward owners and colleagues and to a general work-related stress. Stress associated with detachment was significantly related to more general work-related stress and anxiety and not to decreased empathy.

### 3.5. Questionnaire: Empathy

**Figure 4:**
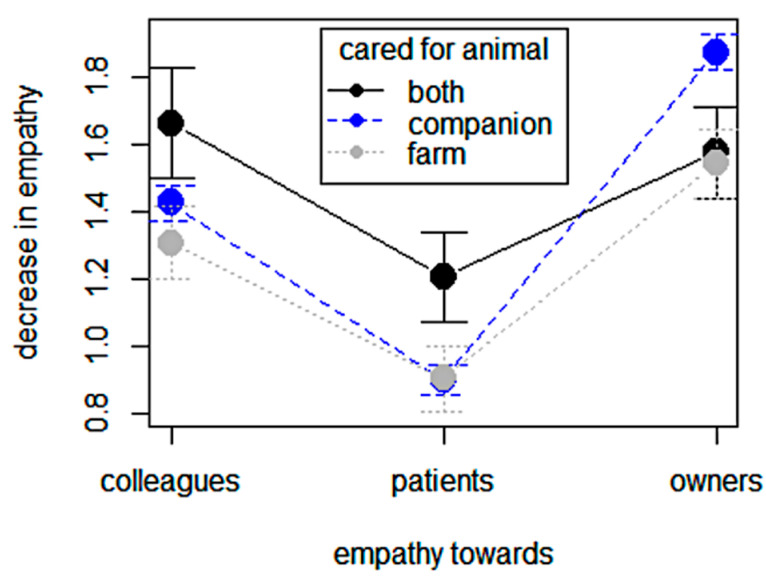
Empathy and cared-for animals: changes in empathy toward colleagues, clients, and animal patients in relation to the type of animal cared for (companion animals, farm animals, and companion plus farm animals).

Changes in empathic relationships with clients, colleagues, and animal patients are depicted in Figure 4, which shows that veterinarians reported a rather slight decrease in empathy toward animals (0.93 ± 1.1) and a more noticeable one toward colleagues (1.42 ± 1.2), especially toward animal owners in general (1.78 ± 1.2). There were significant differences with respect to the objects of empathy (F [2,1396] = 172.6, *p* < 0.001) and, in particular, in interaction with the animal being cared for (F [4,1396] = 5.52, *p* = 0.001). The decrease in empathy toward animal patients was the lowest in both categories of cared-for animals; the decrease in empathy toward owners was significantly more marked among veterinarians caring for companion animals, while that toward colleagues was intermediate and slightly higher in veterinarians caring for both companions and farm animals. Gender had no effect on empathy (F [2,2082] = 0.869, *p* = 0.351), while years of experience seemed to exert a weak protective effect in interaction with the animal cared for (b1 = −0.022, R2 = 0.049, F [1,2077] = 108.81, *p* < 0.001). The decrease in empathy toward colleagues and owners was lower among veterinarians with more years of experience yet more pronounced among vets caring for both companions and farm animals compared to companion and farm vets.

### 3.6. Questionnaire: Burnout

The mean scores for each MBI dimension, doubled to evaluate the animal patient and the human client as sources of burnout, were calculated and are reported in Table 2. As the table shows for each dimension of burnout, the mean scores were higher for the relationship with the client than for that with the animal, indicating that human clients were the major source of both emotional exhaustion and depersonalization. Medium and high levels of animal-related emotional exhaustion were reported by 25.6% and 42.6% of the veterinarians, whereas 19.6% and 59.5% reported medium and high client-related emotional exhaustion, respectively. Medium and high animal-related levels of depersonalization were reported by 28.5 and 23.6 of the veterinarians (more than 50% of respondents), whereas 22.7% and 58% of vets (more than 80%) reported medium and high levels of depersonalization related to clients. Finally, the reported level of personal accomplishment was very low.

**Figure 5:**
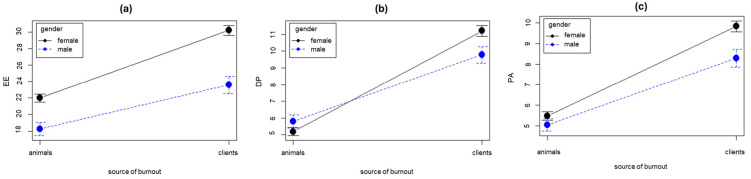
Gender differences in burnout: (**a**) animal- and client-related emotional exhaustion (EE); (**b**) animal- and client-related depersonalization (DP); (**c**) animal- and client-related personal accomplishment (PA).

**Figure 6:**
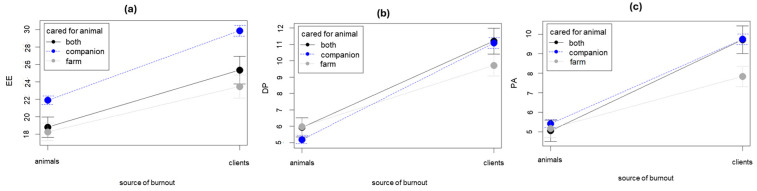
Cared-for animal and burnout: (**a**) animal- and client-related emotional exhaustion (EE); (**b**) animal- and client-related depersonalization (DP); (**c**) animal- and client-related personal accomplishment (PA).

The gender × source of burnout effects (Figure 5) and the cared-for animal × source of burnout effects (Figure 6) on the MBI dimensions were assessed with mixed repeated measures 2 × 2 ANOVA. All the client-related dimensions were significantly greater than those related to the animal; this effect was medium for the emotional exhaustion dimension (EE: F [1,696] = 1829.67, *p* < 0.001, d = 0.61) and strong for the depersonalization dimension (DP: F [1,703] = 829.28, *p* < 0.001, d = 0.84) and the (reduced) personal accomplishment dimension (PA: F [1,703] = 658.67, *p* < 0.001, d = 0.810). Gender had a significant main effect on emotional exhaustion (EE: F [1,696] = 25.57, *p* < 0.001, d = 0.49) and personal accomplishment (PA: F [1,696] = 5.95, *p* = 0.015, d = 0.17), but not on depersonalization (DP: F [1,696] = 0.719, *p* = 0.396, d = 0.07), with women showing greater patient- and client-related burnout than men in both the EE and PA dimensions. However, gender showed significant interactions with the source of burnout in all dimensions (EE: F [1,696] = 57.56, *p* < 0.001; DP: F [1,696] = 24.41, *p* < 0.001; PA: F [1,696] = 10.42, *p* < 0.001); the difference between women and men in DP and PA was absent when the source was the animal (*p* = 0.895, d = 0.11 and *p* = 0.911, d = 0.09, respectively) but significant when the source was the client (*p* = 0.041, d = 0.20 and *p* = 0.005, d = 0.26, respectively); in EE, the significant gender difference resulting from the relationship with animals (*p* = 0.004, d = 0.35) increases when derived from the relationship with clients (*p* < 0.001, d = 0.47).

The cared-for animal (companion vs. farm vs. both) had a significant principal effect on emotional exhaustion (EE: F [2,701] = 9.953, *p* < 0.001, d = 0.61) but not on depersonalization (DP: F [2,701] = 0.338, *p* < 0.001, d = 0.713) nor on personal accomplishment (PA: F [2,701] = 2.451, *p* = 0.087, d = 0.810). The EE resulting from caring for companion animals was significantly greater, and this difference among groups was even greater for the emotional exhaustion resulting from the relationship with clients.

The interactions between cared-for animals and the source of burnout (Table 3) were significant for all burnout dimensions (EE: F [2,701] = 19.376, *p* < 0.001; DP: F [2,701] = 9.301, *p* < 0.001; RP: F [1,696] = 8.711, *p* < 0.001, Figure 6). The emotional exhaustion resulting from the relationship with the animals was significantly greater for those who cared for farm animals (F [2;701] = 7.783, *p* = 0.001), and this difference was further amplified when considering the relationship with the client (F [2;701] = 12.0, *p* < 0.001). Personal accomplishment (PA) and depersonalization (DP) deriving from the relationship with the animal were not significantly different depending on the cared-for animal (F [2,701] = 1.479, *p* = 0.228 and F [2;701] = 0.300, *p* = 0.741, respectively); only the reduced personal accomplishment deriving from the relationship with the clients saw the veterinarians caring for both companion and farm animals significantly penalized (F [2,701] = 1.855, *p* = 0.157 and F [2;701] = 5.222, *p* = 0.006, respectively).

### 3.7. Moral Distress and Burnout

The correlations between moral distress and the other dimensions of work-related distress were evaluated using Pearson’s r coefficient and are reported in Table 4 Due to the large sample size, almost all correlations, even weak or negligible ones, were statistically significant; however, work-related anxiety, stress, and EE showed also interpretively noteworthy positive correlations with moral distress.

Since anxiety, stress, and emotional exhaustion are highly correlated dimensions, partial correlations were used to verify that their relationships with moral distress was not spurious. Indeed, all their correlations with moral distress strongly decreased (Table 5), suggesting a strong overlap, i.e., a great covariance among these variables, probably reflecting the unavoidable intertwining between anxiety, emotional exhaustion, and stress/distress.

Interestingly, the rather strong correlation between moral distress and emotional exhaustion deriving from the relationship with animals disappeared after controlling for the effect of emotional exhaustion deriving from a conflictual relationship with the client, the real relationship being between moral distress and EE clients, which in fact remained significant, even if weak, controlling for the effect of EE.

## 4. Discussion

Moral distress has received attention in human healthcare professions, due to its impact on care providers’ well-being and work performance and its relationship with burnout [11,16,17]. Despite the incidence of psychological distress and burnout in animal healthcare professions and the evidence that ethical challenges and conflicts are common in veterinary practice [5,6,7,9], moral distress and its relationship with burnout have been poorly investigated. To our knowledge, this is the first study assessing moral distress in Italian veterinarians working with companion animals, farm animals, or both and evaluating the possible relationship between moral distress and burnout suggested in previous studies [16,48].

The questionnaire developed to evaluate moral distress showed good consistency and reliability and, therefore, may represent a useful tool for future studies in Italian veterinarians. The factors extracted by factorial analysis confirm that moral distress has a complex and multifaceted nature and multiple root causes [9,51] and show that our questionnaire retains the key components, and the three levels of root causes described in previous studies on animal care providers [6,7,9,51].

The first factor—Conflict with the owner/farmer—encompasses situations characterized by conflicts related to either the animal treatment/care or the veterinarian’s experience of knowing what would be morally right/better to do without being able to act accordingly [6,13]. Interestingly, the problematic relationships with clients, either owners or farmers, also emerged with regard to empathy and burnout; vets’ empathy toward clients decreased with time and the client-related dimensions of burnout were the more relevant.

The second factor—Relationships with colleagues/superiors and Lack of support—encompasses both unit and system causes of moral distress, such as working with colleagues considered not competent enough or providing inadequate therapies, disagreement on how to treat shared cases and not acting or speaking up when faced with unethical or professionally incorrect behaviors for fear of “being penalized” by colleagues, clients, or one’s own structure, and experiencing a lack of administrative action or support for problems that compromised patient care.

Finally, the Unshared requests for the euthanasia factor encompasses requests/pressures to practice or not to practice euthanasia for non-shared reasons. This issue has been initially underlined by Rollin [33] and continues to be considered a core aspect of veterinarians’ moral distress [6,34,35,36].

In line with previous findings [6,9], the level of work-related stress/anxiety was high (34.9% of vets declared to be very stressed, 32.3% reported to be highly stressed, and 56.8% reported to be very or extremely anxious); women were more anxious and stressed than men, and there was a modulating effect depending on years of experience.

Despite high work-related stress/anxiety, veterinarians reported rather low moral distress (mean composite scores below 6 in a range from 0 to 16). Overall, mean scores for intensity and frequency of morally distressing situations suggest that, in our sample of veterinarians, various situations occurred rarely or just sometimes; similarly, the level of experienced stress varied in intensity depending on the situation but, in general, was slight or moderate. The most morally distressing situations were those related to conflicts with owners or farmers regarding the patient’s therapy, being unable to do the right thing, being asked to do things that were considered “the wrong thing to do” or not in the best interest of the patient animal, and euthanasia. As, to our knowledge, this is the only study on moral distress in Italian veterinarians, it is difficult and premature to draw conclusions from this finding.

Different possible explanations could account for this relatively low level of moral distress in the presence of high-level work-related stress and anxiety. As suggested by [6], it is possible that ethical conflicts and the resulting moral distress, although common in contemporary veterinary practice, were not clearly identified or labeled by veterinarians as such. This inability to properly label and recognize moral distress might have led veterinarians to consider conflictual situations as part and parcel of their practice and thus as a source of work-related stress. It is also possible that a process of “habituation” occurred in veterinarians, rendering them less sensitive to ethical issues, as may happen in human healthcare professions. Another possibility could be that, as observed in human healthcare professions, work commitment and a strong professional identity might be of help in the management and mitigation of moral distress in animal healthcare professionals through the development of moral resilience, i.e., the capacity to respond positively to the distress and adversity caused by an ethically complex situation [53,54].

The negative correlation between years of experience and moral distress could depend either on habituation to moral conflicts and moral disengagement or on vets learning through experience to address and cope with ethical problems in clinical practice developing resilience. In our study, the role of coping strategies and moral resilience in the face of morally challenging situations was not investigated, and further studies are needed.

Whether a situation is perceived as morally challenging or not depends on factors such as the veterinarian’s own viewpoints about ethics and the role they ascribe themselves; moreover, experiences, background, feelings, beliefs, and judgments may play a role [2,4,12,55,56,57,58]. These aspects were not considered in the present study and deserve to be addressed in the future along with the utility of addressing moral distress in veterinary school curricula and veterinary practice, providing tools for acknowledging it and coping with it [6,9,59].

Another interesting finding is the effect of the type of animal cared for on moral distress and the level of work-related stress and anxiety; veterinarians working with companion animals or companion plus farm animals reported a significantly higher level of moral distress compared to colleagues working with farm animals. Similarly, the level of work-related stress and anxiety was significantly higher in companion animal vets than in farm vets and, to a lesser extent, than in vets dealing with both typologies. The way in which animals are considered and cared for strongly depends on factors such as attitudes toward them, the perception of their utility and instrumental value, and the role a veterinarian assigns to himself/herself [4,60,61,62,63]. These factors could account for differences in both moral distress and work-related distress among the two types of veterinarians. However, the differences in moral distress that emerged in this study need further investigation, as other aspects could probably be involved.

The evidence that, regardless of the type of animal cared for, women reported significantly higher levels of moral distress than men, even after controlling for work experience, is in line with the literature on moral distress [6,9,50] and deserves consideration given the process of feminization underway in Italy, especially among small animal veterinarians.

The items assessing veterinarians’ perceived changes in compassion/empathy toward clients/colleagues and animal patients revealed that the decline in empathy toward animals was low compared to that toward colleagues and especially clients and that the decrease in empathy was greater in professionals working with companion animals. Empathy is considered a key component of high-quality care in both human and animal health professions, and veterinarians play a key role in animal welfare [62,64]. The human–animal bond is complex and multifaceted in its nature, and in general, the bond between human caregiver and their companion animals is psychologically intense and relevant for both partners and has been described as an attachment [61]; therefore, companion animals’ vets may be required to care for both their patients and human clients, showing sensitivity and empathy toward both [65,66]. This additional effort of “emotional attunement” may involve higher levels of stress and empathic over-arousal, favoring emotional detachment or even compassion fatigue burnout [67,68,69].

Half of the veterinarians reported experiencing quite often that they are “just doing their job” and considered this emotional detachment as related mainly to a decline in empathy toward animal patients and to general work-related stress, independent of the animal they cared for; women were more distressed by this condition of detachment than men. Emotional detachment has been reported in previous studies, and recently, Moses et al. [6] found that 41.5% and 17.7% of US veterinarians experienced this emotional condition sometimes and often, respectively.

In human healthcare professions, persistent moral distress is a major cause of emotional exhaustion, depression, and burnout, leading to indifference in morally challenging situations [16,27]. The modified version of the Italian MBI scale [50] assessing the impact of clients and animals on emotional exhaustion, depersonalization, and personal accomplishment showed that client-derived levels of burnout significantly exceeded animal-derived ones in all the burnout dimensions. The client-derived burnout was greater than the animal-derived burnout in both genders, yet women reported higher emotional exhaustion and depersonalization and a lower level of personal accomplishment; in addition, women reported a higher level of emotional exhaustion related to the animal patient.

These findings support previous evidence on veterinarians [28,29,30], showing that burnout is a problem in terms of emotional exhaustion, depersonalization, and low personal accomplishment and that female vets are at a greater risk of psychological distress than males; in addition, they indicate that veterinarians caring for companion animals experience a greater animal and client-related emotional exhaustion than those caring for farm animals or both animal types.

A relationship between moral distress and the different dimensions of burnout emerged: higher levels of moral distress corresponded to higher values of emotional exhaustion and depersonalization and, to a lesser extent, lower professional fulfillment. Further studies are needed to clarify whether moral distress aggravates burnout or, conversely, exhaustion and detachment amplify the perception of moral conflicts.

The finding that professionals caring for companion animals reported higher levels of moral distress, a greater decrease in empathy (especially toward clients), and a significantly higher client-related emotional exhaustion compared to their colleagues working with farm animals needs further investigation and underlines the importance of implementing in both veterinary education and practice the knowledge and skills to better manage relationships and communication with human clients and colleagues; this is an aspect that is often underestimated or even absent in veterinary education, at least in Italy.

## 5. Conclusions

Despite the incidence of psychological distress and burnout in veterinarians and the evidence that ethical challenges and conflicts are common in veterinary practice, moral distress and its relationship with burnout have been scarcely investigated. Through an online survey, we compared Italian veterinarians caring for companion animals, farm animals, or both, evaluating their levels of moral distress, work-related stress/anxiety, empathy, and burnout. The moral distress questionnaire showed good consistency and reliability and could be a useful tool for future studies. The four factors extracted by factorial analysis confirmed the complex and multifaceted nature of moral distress and its multiple root causes. The finding that moral distress was rather low compared to general work-related stress/anxiety needs further investigation, as different explanations are possible. The evidence that professionals caring for companion animals reported higher levels of work-related distress, anxiety, and moral distress, a greater decrease in empathy toward clients, and a significantly higher level of client-related burnout needs further investigation, as different explanations are possible. Interestingly, however, in general, colleagues, and especially clients, were the main source of psychological distress, burnout, and empathy decline in veterinarians. The greater sensitivity to moral distress and burnout found in women should not be overlooked given the progressive feminization of the veterinary profession in Italy and worldwide.

## Figures and Tables

**Table 1:** Moral Distress items divided by factor are reported. For each item, frequency of occurrence, stress intensity, and total score are reported. Items not well saturated and excluded from factor analysis are also reported.

MD Items	Frequency (Mean ± SD)	Stress Intensity (Mean ± SD)	Overall (Mean ± SD)
Factor I—Conflict with owner/farmer			
25. Has it happened to you that the owner/farmer did not follow the recommended therapy correctly?	2.31 ± 0.76	2.26 ± 0.96	5.38 ± 3.42
3. Have you ever felt like you were unable to do the “right thing to do”?	1.97 ± 0.87	2.37 ± 0.87	5.09 ± 2.97
26. Did it happen that the owner/breeder made discriminations based on the type of animal (for example, sex, age…), when deciding to take care of it?	2.1 ± 1.1	2.1 ± 1.2	4.87 ± 3.93
6. Have you ever felt in conflict or upset because an animal owner/farmer refused to do what you think is in the “best interest of your patient”?	2.0 ± 0.79	2.21 ± 0.89	4.82 ± 2.90
12. Have you been in a situation where the owner/farmer was unwilling to pay for the therapy prescribed to the animal?	2.1 ± 0.97	2.1 ± 1.0	4.66 ± 3.25
1. How often do you have a conflict of opinion with the owners/farmers of the animals you care for on how to proceed in the treatment of their animals?	1.94 ± 0.82	2.18 ± 0.85	4.51 ± 2.77
2. Have you been asked to do anything during your clinical practice that you considered “the wrong thing to do”?	1.76 ± 0.82	2.33 ± 0.94	4.5 ± 2.75
8. Have owners/farmers ever put obstacles to the animals following the therapies you recommended?	1.92 ± 0.85	1.93 ± 0.95	4.17 ± 2.89
10. Have you ever been asked to do things that were not within your competency, for financial or other reasons?	1.69 ± 1.2	1.77 ± 1.17	4.01 ± 2.89
7. Have you ever had the feeling that you give more importance to the needs of the owners/breeders, rather than to the needs of your animal patients?	1.73 ± 1.1	1.9 ± 1.1	3.87 ± 3.14
5. Have you handled situations in which the animal owner/farmer asked for a treatment that you felt was unnecessary for the animal?	1.67 ± 0.84	1.74 ± 0.96	3.32 ± 2.63
4. How often do you find yourself recommending euthanasia to animal owners/farmers?	1.84 ± 0.63	1.69 ± 0.94	3.12 ± 2.10
24. Have you ever been unable to practice euthanasia for reasons that you did not agree with?	1.14 ± 0.87	1.86 ± 1.40	2.92 ± 2.75
Factor II—Relationships with colleagues/superiors			
17. Have you ever worked with colleagues that you considered not competent enough?	1.51 ± 0.99	1.77 ± 1.15	3.38 ± 2.96
13. Have you ever found yourself working with colleagues who in your opinion offered inadequate therapies?	1.55 ± 0.93	1.55 ± 0.93	3.33 ± 2.83
20. Have you ever not said or done what you considered ethically and professionally correct for fear of being “penalized” by colleagues, clients, or the structures you worked for?	0.73 ± 0.98	1.1 ± 1.3	1.78 ± ±2.78
11. Have you ever disagreed with other colleagues in veterinary medicine regarding the best way to handle a shared case?	1.57 ± 0.85	1.68 ± 0.90	1.57 ± 0.85
19. Have you ever been pressured by colleagues or superiors not to intervene when you knew that a colleague had made a clinical error but would not admit it?	0.40 ± 0.77	0.64 ± 1.14	0.99 ± 2.11
Factor III—Legal context/unsupportive Professional Institutions			
9. Have you ever suspected that there was mistreatment/abuse of the animal you were caring for?	1.1 ± 0.77	1.99 ± 1.26	2.74 ± 2.28
14. Have you ever witnessed the violation of standard procedures or ethical principles while not feeling adequately supported to report that violation?	0.98 ± 1.1	1.42 ± 1.35	2.50 ± 3.1
15. Have you ever experienced a lack of appropriate support and intervention following reports that animal welfare was not respected/compromised?	0.89 ± 1.1	1.20 ± 1.29	2.18 ± 2.96
18. Have you ever had to implement therapies requested by owners/farmers, even if you did not agree, for fear of legal disputes?	0.40 ± 0.69	0.74 ± 1.18	0.95 ± 1.81
Factor IV—Unshared requests for euthanasia			
22. Have you ever received requests to practice euthanasia that you felt were inappropriate?	1.32 ± 0.84	2.28 ± 1.41	3.67 ± 3.15
23. Have you ever practiced euthanasia for reasons you disagreed with?	0.41 ± 0.62	1.04 ± 1.53	1.18 ± 1.93
Items with unsatisfying factorial loadings 16. Have you ever witnessed an animal receiving insufficient/wrong therapy due to a lack of the necessary resources/equipment?	1.52 ± 1.0	1.88 ± 1.13	3.57 ± 2.99
21. Have you ever talked to your colleagues about situations of moral conflict that occurred in your work?	1.71 ± 1.13	1.33 ± 1.10	2.98 ± 3.15
Items addressed only to veterinarians working with farm animals *			
Have you ever received requests to put an animal down that you felt were inappropriate?	0.84 ± 0.89	1.38 ± 1.48	2.03 ± 2.72
Have you ever been unable to put an animal down when you thought that it was necessary?	0.83 ± 0.82	1.39 ± 1.40	1.97 ± 2.30
Have you ever had to put an animal down even if you did not agree with it?	0.41 ± 0.72	0.78 ± 1.35	1.11 ± 2.16

* Items addressed only to veterinarians working with farm animals were not included in the factorial analysis.

**Table 2:** Mean scores (±SD) for each of the 3 subscales of the MBI related to the interaction with the animal and the client. The number and % of participants reporting low, medium, and high burnout levels are reported.

Burnout	Emotional Exhaustion		Depersonalization		Personal Accomplishment	
	Animal	Client	Animal	Client	Animal	Client
Mean ± SD	20.94 ± 10.87	28.31 ± 14.35	5.39 ± 5.36	10.86 ± 7.31	5.34 ± 0.52	9.41 ± 5.99
Burnout level: N (%)						
- Low	224 (31.6)	147 (20.9)	337 (47.9)	136 (19.3)	--	--
- Medium	180 (25.6)	138 (19.6)	201 (28.5)	160 (22.7)	--	2 (0.3)
- High	300 (42.6)	419 (59.5)	166 (23.6)	408 (58.0)	704 (100)	702 (99.7)

**Table 3:** MBI scores: mean scores (±SD) for each of the 3 subscales of the MBI related to the interaction with the animal and the client.

Animal Type	EE			DP			PA		
	Patients	Clients	Overall	Patients	Clients	Overall	Patients	Clients	Overall
Companion	21.9 ± 10.8	29.8 ± 14.1	25.9 ± 13.1	5.2 ± 5.4	11.1 ± 7.4	8.1±7.1 5.4±4.4 9.7±5.9 7.6±5.7	5.4 ± 4.4	9.7 ± 5.9	7.6 ± 5.7
Farm	18.2 ± 11.3	23.5 ± 14.9	20.9 ± 13.4	5.9 ± 5.6	9.7 ± 7.2	7.8 ± 6.7	5.2 ± 5.1	7.8 ± 5.8	6.5 ± 5.6
Both	18.8 ± 9.9	25.4 ± 13.3	22.07 ± 12.2	5.9 ± 4.9	11.2 ± 6.6	8.6 ± 6.3	5.9 ± 4.6	9.7 ± 5.9	7.4 ± 5.8

**Table 4:** Zero-order correlations between moral distress and other dimensions of work-related distress.

	Burnout—Animals			Burnout—Owners		
	EE	DP	PA	EE	DP	PA
Moral distress	0.543 **	0.308 **	0.162 **	0.572 **	0.481 **	0.317 **
	Decreased empathy			Anxiety	Work-related stress	
	Patients	Owners	Colleagues			
Moral distress	0.202 **	0.376 **	0.322 **	0.562 **	0.584 **	

** *p* < 0.01: Holm’s correction for multiple correlations.

**Table 5:** First-order correlations between moral distress and other dimensions of work-related distress.

Correlation Between Moral Distress and	Controlling for			
	Anxiety	Work-Related Stress	EE Animals	EE Owners
Work-related anxiety	--	0.209 **	0.305 **	0.267 **
Work-related stress	r = 0.301 **	--	0.311 **	r = 0.274 **
EE animals	0.305 **	0.225 **	--	−0.04
EE owners	r = 0.331 **	r = 0.261 **	0.206 **	--

** *p* < 0.01.

## Data Availability

The data of the study are available at the following link: https://univpr-my.sharepoint.com/:x:/g/personal/annalisa_pelosi_unipr_it/EdNbEgwVnWdKsdPynMaiO4QBrh_T1PfA9che-ly5swGfqA?rtime=IQnyw9Ug3Ug&nav=MTVfezAwMDAwMDAwLTAwMDEtMDAwMC0wMDAwLTAwMDAwMDAwMDAwMH0 (accessed on 1 December 2024).

## Supplementary Materials

The following supporting information can be downloaded at: https://www.mdpi.com/article/10.3390/ani14243691/s1. S1—Questionnaire.
