# Supplementary material for: Willing but Unable: Moral Distress and Burnout in Italian Veterinarians Working with Companion and Farm Animals
Source: Animals (Basel). 2024 Dec 20;14(24):3691. doi: 10.3390/ani14243691 (PMC11672766; doi:10.3390/ani14243691)
Supplement: Supplementary file 1 [file animals-14-03691-s001.zip › animals-3341176-supplementary.pdf]

Willing but unable: a study on moral distress and burnout in Italian veterinarians

MORAL DISTRESS & BURNOUT QUESTIONNAIRE

NOTE: The items marked with 1 were answered only by veterinarians working with pets, those marked with 2 only by veterinarians working with farm animals.

A. GENERAL INFORMATION:

Gender: □ F □ M □ Non binary

Age: ……

Number of persons in the household (including yourself): ……..

Did you grow up with domestic animals at home? □ Yes □ No
If Yes, what type of animals? **……………**

Do you presently have domestic animal at home? □ Yes □ No
If Yes, what type of animals?........................

B. WORK ACTIVITY:

You have practiced veterinary medicine for .... **years**

Do you have a specialization in a field of veterinary medicine?
□ No □ Yes (please specify): …..

In the past 5 years, how many times have you changed workplace or or working modality? …

In which Italian area do you currently practice your profession?

□ North West □ North East □ Centre □ South □ Islands

Do you work alone? □ Yes □ No

If you work with others, how many collaborators do you have, approximately?

- Vets: ………
- Non vets: ……

Which domestic animals do you take care of?

□ Only companion animals (please specify): …

□ Only farm animals (please specify): …

□ Both companion and farm animals (please specify): …

C. PROFESSIONAL EXPERIENCES & MORAL DISTRESS

1. How often do you have a conflict of opinion with the owners/farmers of the animals you care for on how to proceed in the treatment of their animals?
□ Never □ Rarely □ Sometimes □ Often □ Very often

If it has happened to you at least once, how much stress did it cause you?
□ None □ Mild □ Moderate □ Strong □ Extreme

2. Have you been asked to do anything during your clinical practice that you considered "the wrong thing to do"?
□ Never □ Rarely □ Sometimes □ Often □ Very often

If it has happened to you at least once, how much stress did it cause you?
□ None □ Mild □ Moderate □ Strong □ Extreme

3. Have you ever felt like you were unable to do the "right thing to do"?
□ Never □ Rarely □ Sometimes □ Often □ Very often

If it has happened to you at least once, how much stress did it cause you?
□ None □ Mild □ Moderate □ Strong □ Extreme

4-1. Have you ever received requests to practice euthanasia that you felt were inappropriate?
□ Never □ Rarely □ Sometimes □ Often □ Very often

If it has happened to you at least once, how much stress did it cause you?
□ None □ Mild □ Moderate □ Strong □ Extreme

4-2. Have you ever received requests to put an animal down that you felt were inappropriate?
□ Never □ Rarely □ Sometimes □ Often □ Very often

If it has happened to you at least once, how much stress did it cause you?
□ None □ Mild □ Moderate □ Strong □ Extreme

5-1. Have you ever practiced euthanasia for reasons you disagreed with?
□ Never □ Rarely □ Sometimes □ Often □ Very often

If it has happened to you at least once, how much stress did it cause you?

□ None □ Mild □ Moderate □ Strong □ Extreme

5-2. Have you ever had to put an animal down even if you didn't agree with it?
□ Never □ Rarely □ Sometimes □ Often □ Very often

If it has happened to you at least once, how much stress did it cause you?
□ None □ Mild □ Moderate □ Strong □ Extreme

6. How often do you find yourself recommending euthanasia to animal owners/farmers?
□ Never □ Rarely □ Sometimes □ Often □ Very often

If it has happened to you at least once, how much stress did it cause you?
□ None □ Mild □ Moderate □ Strong □ Extreme

7. Have you ever been unable to practice euthanasia for reasons that you did not agree with?
□ Never □ Rarely □ Sometimes □ Often □ Very often

If it has happened to you at least once, how much stress did it cause you?
□ None □ Mild □ Moderate □ Strong □ Extreme

8. Have you handled situations in which the animal owner/ farmer asked for a treatment that you felt was unnecessary for the animal?
□ Never □ Rarely □ Sometimes □ Often □ Very often

If it has happened to you at least once, how much stress did it cause you?
□ None □ Mild □ Moderate □ Strong □ Extreme

9. Have you ever felt in conflict or upset because an animal owner/farmer refused to do what you think is in the "best interest of your patient"?
□ Never □ Rarely □ Sometimes □ Often □ Very often

If it has happened to you at least once, how much stress did it cause you?
□ None □ Mild □ Moderate □ Strong □ Extreme

10. Have you ever had the feeling that you give more importance to the needs of the owners/farmers rather than to the needs of your animal patients?
□ Never □ Rarely □ Sometimes □ Often □ Very often

If it has happened to you at least once, how much stress did it cause you?
□ None □ Mild □ Moderate □ Strong □ Extreme

11. Have owners/farmers ever put obstacles to the animals following the therapies you recommended?
□ Never □ Rarely □ Sometimes □ Often □ Very often

If it has happened to you at least once, how much stress did it cause you?
□ None □ Mild □ Moderate □ Strong □ Extreme

12. Have you ever suspected that there was mistreatment/abuse of the animal you were caring for?
□ Never □ Rarely □ Sometimes □ Often □ Very often

If it has happened to you at least once, how much stress did it cause you?
□ None □ Mild □ Moderate □ Strong □ Extreme

13. Have you ever felt stressed because of your job?
□ Never □ Rarely □ Sometimes □ Often □ Very often

14. Have you ever felt anxious because of your job?
□ Never □ Rarely □ Sometimes □ Often □ Very often

15. Have you ever been asked to do things that were not within your competency for financial or other reasons?
□ Never □ Rarely □ Sometimes □ Often □ Very often

If it has happened to you at least once, how much stress did it cause you?
□ None □ Mild □ Moderate □ Strong □ Extreme

16. Have you ever disagreed with other colleagues in veterinary medicine regarding the best way to handle a shared case?
□ Never □ Rarely □ Sometimes □ Often □ Very often

If it has happened to you at least once, how much stress did it cause you?
□ None □ Mild □ Moderate □ Strong □ Extreme

17. Have you been in a situation where the owner/farmer was unwilling to pay for the therapy prescribed to the animal?
□ Never □ Rarely □ Sometimes □ Often □ Very often

If it has happened to you at least once, how much stress did it cause you?
□ None □ Mild □ Moderate □ Strong □ Extreme

18. Have you ever found yourself working with colleagues who in your opinion offered inadequate therapies?
□ Never □ Rarely □ Sometimes □ Often □ Very often

If it has happened to you at least once, how much stress did it cause you?
□ None □ Mild □ Moderate □ Strong □ Extreme

19. Have you ever witnessed the violation of standard procedures or ethical principles while not feeling adequately supported to report that violation?
□ Never □ Rarely □ Sometimes □ Often □ Very often

If it has happened to you at least once, how much stress did it cause you?
□ None □ Mild □ Moderate □ Strong □ Extreme

20. Have you ever experienced a lack of appropriate support and intervention following reports that animal welfare was not respected/compromised?
□ Never □ Rarely □ Sometimes □ Often □ Very often

If it has happened to you at least once, how much stress did it cause you?

□ None □ Mild □ Moderate □ Strong □ Extreme

21. Have you ever witnessed an animal receiving insufficient/wrong therapy due to a lack of the necessary resources/equipment?
□ Never □ Rarely □ Sometimes □ Often □ Very often

If it has happened to you at least once, how much stress did it cause you?
□ None □ Mild □ Moderate □ Strong □ Extreme

22. Have you ever worked with colleagues that you considered not competent enough?
□ Never □ Rarely □ Sometimes □ Often □ Very often

If it has happened to you at least once, how much stress did it cause you?
□ None □ Mild □ Moderate □ Strong □ Extreme

23. Have you ever had to implement therapies requested by owners/farmers, even if you did not agree, for fear of legal disputes?
□ Never □ Rarely □ Sometimes □ Often □ Very often

If it has happened to you at least once, how much stress did it cause you?
□ None □ Mild □ Moderate □ Strong □ Extreme

24. Have you ever been pressured by colleagues or superiors not to intervene when you knew that a colleague had made a clinical error but would not admit it?
□ Never □ Rarely □ Sometimes □ Often □ Very often

If it has happened to you at least once, how much stress did it cause you?
□ None □ Mild □ Moderate □ Strong □ Extreme

25. Have you ever not said or done what you considered ethically and professionally correct for fear of being "penalized" by colleagues, clients or the structures you worked for?
□ Never □ Rarely □ Sometimes □ Often □ Very often

If it has happened to you at least once, how much stress did it cause you?
□ None □ Mild □ Moderate □ Strong □ Extreme

26. Have you ever talked to your colleagues about situations of moral conflict that occurred in your work?
□ Never □ Rarely □ Sometimes □ Often □ Very often

If it has happened to you at least once, how much stress did it cause you?
□ None □ Mild □ Moderate □ Strong □ Extreme

27. Do you feel that your capacity to feel compassion and empathy for your animal patients decreased during your practice?
□ Per nulla □ Poco □ Abbastanza □ Molto □ Moltissimo

28. Do you feel that your empathy for colleagues decreased during your practice?
□ Per nulla □ Poco □ Abbastanza □ Molto □ Moltissimo

29-1. Do you feel that your capacity to feel empathy for animal owners decreased during your practice?
□ Per nulla □ Poco □ Abbastanza □ Molto □ Moltissimo

29-2. Do you feel that your capacity to feel empathy for farmers decreased during your practice?
□ Per nulla □ Poco □ Abbastanza □ Molto □ Moltissimo

30. How often do you feel that you are 'just' doing your job?
□ Never □ Rarely □ Sometimes □ Often □ Very often

31. Did it happen to you that the owner/farmer did not carry out the indicated therapy correctly?
□ Never □ Rarely □ Sometimes □ Often □ Very often

If it has happened to you at least once, how much stress did it cause you?
□ None □ Mild □ Moderate □ Strong □ Extreme

32. Did it happen that the owner/ farmer made discriminations based on the type of animal (for example, sex, age...), when deciding to take care of it?
□ Never □ Rarely □ Sometimes □ Often □ Very often

If it has happened to you at least once, how much stress did it cause you?
□ None □ Mild □ Moderate □ Strong □ Extreme

D. MASLACH BURNOUT INVENTORY (MBI) -MODIFIED VERSION (MBI-VET)

	**Never**	**A few times per year**	**Once a month or less**	**A few times per month**	**Once a week**	**A few times per week**	**Every day**
1) I feel emotionally exaused by my work.	□	□	□	□	□	□	□
2) I feel exhausted at the end of a working day.	□	□	□	□	□	□	□
3) I feel tired when I get up in the morning and have to face another day at work.	□	□	□	□	□	□	□
4) I can easily understand what my clients are feeling.	□	□	□	□	□	□	□
4a) I can easily grasp/understand what animals are feeling	□	□	□	□	□	□	□

	Never	A few times per year	Once a month or less	A few times per month	Once a week	A few times per week	Every day
5) I feel I treat some clients as if they were objects	□	□	□	□	□	□	□
5a) I feel I treat some animals as if they were objects	□	□	□	□	□	□	□
6) I feel that working all day with people is a strain for me.	□	□	□	□	□	□	□
6a) I feel that working all day with animals is really a strain for me.	□	□	□	□	□	□	□
7) I deal with my clients' problems effectively.	□	□	□	□	□	□	□
7a) I deal with animals' problems effectively.	□	□	□	□	□	□	□
8) I feel depleted by my work.	□	□	□	□	□	□	□
9) I feel I'm positively influencing other people's lives through my work.	□	□	□	□	□	□	□
9a) I feel I'm positively influencing animals' lives through my work.	□	□	□	□	□	□	□
10) Since I took this job, I am more callous towards people.	□	□	□	□	□	□	□
10a) Since I took this job, I am more callous towards animals.	□	□	□	□	□	□	□
11) I worry that this work is hardening me emotionally.	□	□	□	□	□	□	□
12) I feel very energetic	□	□	□	□	□	□	□
13) I feel frustrated by my work.	□	□	□	□	□	□	□
14) I feel I'm working too hard on my job.	□	□	□	□	□	□	□
15) I don't really care about what happens to some of my clients.	□	□	□	□	□	□	□

	Never	A few times per year	Once a month or less	A few times per month	Once a week	A few times per week	Every day
15a) I don't really care about what happens to some of my animal patients	□	□	□	□	□	□	□
16) Working in direct contact with people stresses me too much	□	□	□	□	□	□	□
16a) Working in direct contact with animals stresses me too much	□	□	□	□	□	□	□
17) I can easily create a relaxed atmosphere with my clients	□	□	□	□	□	□	□
17a) I can easily create a relaxed atmosphere with my animals	□	□	□	□	□	□	□
18) I feel really satisfied after working with my clients.	□	□	□	□	□	□	□
18a) I feel really satisfied after working with animals.	□	□	□	□	□	□	□
19) I have accomplished many worthwhile things in this job	□	□	□	□	□	□	□
20) I feel at the end of my rope.	□	□	□	□	□	□	□
21) In my work I deal with emotional problems very calmly	□	□	□	□	□	□	□
22) I have the impression that my clients make me responsible for some of their problems	□	□	□	□	□	□	□
22a) I have the impression to cause animals more problems than they would have without my intervention.	□	□	□	□	□	□	□
